# Synaptic vesicle endocytosis deficits underlie GBA-linked cognitive dysfunction in Parkinson’s disease and Dementia with Lewy bodies

**DOI:** 10.1101/2024.10.23.619548

**Published:** 2024-10-23

**Authors:** D J Vidyadhara, David Bäckström, Risha Chakraborty, Jiapeng Ruan, Jae-Min Park, Pramod K. Mistry, Sreeganga. S. Chandra

**Affiliations:** 1 Departments of Neurology, Yale University, CT, USA; 2 Neuroscience, Yale University, CT, USA; 3 Discipline of Neuroscience, Chicago Medical School, Rosalind Franklin University of Medicine and Science, North Chicago, IL, USA; 4 Center for Neurodegenerative Disease and Therapeutics, Chicago Medical School, Rosalind Franklin University of Medicine and Science, North Chicago, IL, USA; 5 Department of Clinical Science, Neurosciences, Umeå University, Sweden; 6 Department of Internal Medicine, Yale University, CT, USA; 7 Van Andel Institute, MI, USA; 8 Program in Cellular Neuroscience, Neurodegeneration and Repair, Yale University, CT, USA

**Keywords:** *GBA*, *SNCA*, Cognitive dysfunction, Parkinson’s disease, Parkinson’s disease with dementia, Dementia with Lewy bodies, Lewy body dementia, snRNA-seq, synaptic vesicle endocytosis, synaptic plasticity, α-synuclein, α-synucleinopathies

## Abstract

*GBA* mutations are major risk factors for Parkinson’s disease (PD) and Dementia with Lewy Bodies (DLB), two common α-synucleinopathies associated with cognitive impairment. Here, we investigated the role of *GBA* mutations in cognitive decline by utilizing Gba L444P mutant mice, SNCA transgenic (tg), and Gba-SNCA double mutant mice. Notably, Gba mutant mice showed early cognitive deficits but no PD-like motor deficits up to 12 months old. Conversely, SNCA tg mice displayed age-related motor deficits but no cognitive abnormalities. Gba-SNCA mice exhibited exacerbated motor deficits and cognitive decline. Immunohistological analysis revealed cortical phospho-α-synuclein pathology in SNCA tg mice, which was exacerbated in Gba-SNCA mice, especially in layer 5 cortical neurons. Significantly, Gba mutant mice did not show α-synuclein pathology. Single-nucleus RNA sequencing of cortices instead uncovered selective synaptic vesicle cycle defects in excitatory neurons of Gba mutant and Gba-SNCA mice, via robust downregulation in gene networks regulating synapse vesicle cycle and synapse assembly. Meanwhile SNCA tg mice displayed broader synaptic changes. Immunohistochemical and electron microscopic analyses validated these findings. Together, our results indicate that Gba mutations, while exacerbating pre-existing α-synuclein aggregation and PD-like motor deficits, contribute to cognitive deficits through α-synuclein-independent mechanisms, likely involving dysfunction in synaptic vesicle endocytosis. Additionally, Gba-SNCA mice are a valuable model for studying cognitive and motor deficits in PD and DLB.

## Introduction:

*GBA* is a major risk allele for Parkinson’s disease (PD) and Dementia with Lewy Bodies (DLB)^1–6^. Both are late-onset neurodegenerative diseases, characterized by the neuronal accumulation of Lewy bodies primarily composed of the presynaptic protein α-synuclein^7^. PD is classified as a movement disorder, although dementia affects around 50% of PD patients within 10 years after symptom onset^7^. DLB is classified as a dementia, in which cognitive decline is generally the first and most predominant symptom^7^. In both diseases, the severity of cognitive deficits is related to loss of autonomy and shorter survival^7^. Significantly, PD patients with *GBA* mutations exhibit greater cognitive deficits and more rapid decline than idiopathic (non-GBA related) PD^1, 8, 9 7^. Cognitive dysfunction in both PD and DLB, which entails visuospatial and memory impairment, as well as executive dysfunction, is strongly correlated with neocortical Lewy body pathology^7^. However, the mechanisms through which *GBA* predisposes to cognitive dysfunction, as well as to developing α-synucleinopathies in general, are not well understood.

Homozygous or biallelic mutations in *GBA* cause the lysosomal storage disorder, Gaucher disease (GD)^10, 11^. Numerous biallelic *GBA* mutations underlie GD, ranging from mild to null mutations^12, 13^. Two prevalent mutations, due to founder effects, are N370S and L444P^12, 13^. GD patients have a 20-fold increased risk of developing PD accompanied by earlier and faster cognitive decline. Heterozygous carriers of *GBA* mutation are at 5-fold increased risk for developing both PD and cognitive dysfunction^1, 2, 8, 9, 14–18^, consistent with a gene dosage effect. In the case of DLB, *GBA* and *SNCA*, the gene for α-synuclein, are top GWAS hits. Interestingly, *GBA* mutations confer an even higher risk of developing DLB^4–6^.

*GBA* encodes glucocerebrosidase 1 (GCase1), a lysosomal hydrolase responsible for breaking down the bioactive lipid glucosylceramide (GlcCer) to glucose and ceramide. In the absence of Gcase1, GlcCer and other glycosphingolipids accumulate. Interestingly, GCase1 deficiency and glycosphingolipid accumulation are also observed in post-mortem brains of patients with sporadic PD and in aging brains^19–22^. Glycosphingolipid accumulation correlates with a higher burden of α-synuclein or Lewy body pathology in brain areas of importance for cognition^20, 22, 23^. Conversely, higher GCase1 enzymatic activity levels in cerebrospinal fluid, early in disease, is negatively correlated with risk of dementia in PD^24^. These genetic, clinical, and epidemiological studies support the importance of clarifying *GBA*’s role in cognitive dysfunction.

The prevailing hypothesis in the field is that *GBA* mutations lead to GCase1 deficiency, which, through a combination of lysosomal dysfunction and glycosphingolipid accumulation, trigger α-synuclein aggregation, resulting in Lewy body formation and consequently, disease associated phenotypes. We and others have shown that glycosphingolipids can directly interact with α-synuclein and promote aggregation *in vitro*^25, 26^. Furthermore, we previously developed long-lived mouse models of GD carrying the Gba N370S and L444P mutations, which exhibits reduced GCase1 activity and accumulation of glycosphingolipids in the liver, spleen, and brain^27^. As GD patients with the L444P mutation have pronounced cognitive deficits^28^, in this study, we conducted a thorough, longitudinal examination of Gba L444P mice, in conjunction with the well-established SNCA tg PD mice that overexpress mutant human α-synuclein, and their crossbreeds, i.e. Gba-SNCA mice. We find that cognitive and motor deficits are dissociable by genotype in these mice, with Gba mutants exhibiting cognitive deficits only, SNCA transgenics motor difficulties only, and Gba-SNCA severe motor and cognitive phenotypes. Through histopathological analyses, we show that Gba L444P mutant mice lack Ser-129-phospho-α-synuclein (pSer129α-syn) pathology but presence of the Gba mutation in Gba-SNCA mice significantly exacerbates this pathology, especially in deep layers of the cortex. Thus, contrary to expectation, cognitive deficits related to Gba mutations may emerge independently of pSer129α-syn pathology. Cortical single-nucleus RNA sequencing (snRNA-seq) analyses of these mice revealed a potential role for synaptic dysfunction, in particular synaptic vesicle endocytosis (SVE) deficits in excitatory neurons contributing to the cognitive decline observed in Gba mutant and Gba-SNCA mice. Synaptic endocytosis deficits are emerging as a central mechanism of PD pathogenesis, especially in rare monogenic forms of PD in causing motor deficits^29, 30^ but these mechanisms have not been linked to *GBA*-mutations and to cognitive dysfunction. This study suggests, in GBA-linked PD and DLB, a role for synaptic endocytosis dysfunction in cognitive deficits and greater α-synuclein pathology burden to amplified motor deficits.

## Results:

### Gba mutation leads to cognitive dysfunction and exacerbates motor deficits in SNCA tg mice:

To determine the relative contributions of *GBA* and *SNCA* to motor and cognitive domains, we performed detailed behavioral analyses of wild-type (WT), Gba, SNCA tg, and Gba-SNCA mouse sex-balanced cohorts. We conducted longitudinal evaluations of motor behavior every 3 months to establish the age of onset and progression of PD-like motor deficits compared to cognitive behavior deficits.

**Figure 1:**
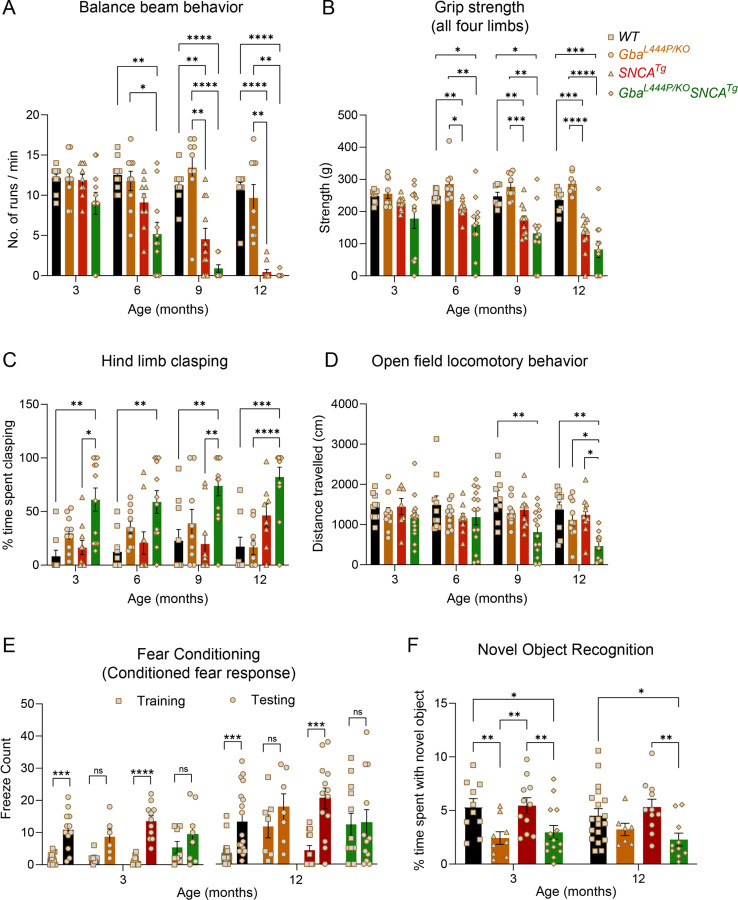
Gba mutation worsens motor deficits in SNCA tg mice and independently leads to cognitive dysfunction. WT, Gba, SNCA tg and Gba-SNCA mice cohorts were evaluated in four motor and two cognitive behavior tests in a longitudinal manner. **A.** Balance beam behavior. **B.** Grip strength of all four limbs. **C.** Hind limb clasping behavior. **D.** Open field locomotory behavior. **E.** Fear conditioning test. **F**. Novel object recognition. n = 9–12 mice for motor behavior and 6–19 for cognitive behavior, both sexes were used. Data are presented as mean ± SEM. ns - not significant; *p < 0.05, **p < 0.01, ***p < 0.001, ****p < 0.0001

Four distinct, complementary assays were used for phenotyping motor deficits: the balance beam, grip strength, hind limb clasping, and open-field locomotion tests. In the balance beam test, a standard test to monitor PD-related motor deficits, mice were required to walk along a narrow beam from a well-lit area to a dark, secure box^30^. The number of runs completed in a minute and the average time per run were used to assess balance beam performance (Fig. 1A, Supp. Fig. 1A, 1D–E). Gba mice consistently performed well on this task, comparable to WT mice, up to 12 months of age. In contrast, SNCA tg mice could perform this task at 3 months but began showing deficits on the balance beam at 6 months, which worsened by 12 months. Notably, Gba-SNCA mice demonstrated exacerbated balance deficits compared to SNCA tg mice, with significant deficits appearing as early as 6 months. By 9–12 months of age, these double mutant mice were severely affected and unable to navigate the balance beam (Fig. 1A, Supp. Fig. 1A, 1D–E). We noted a similar pattern of behavior deficits in grip strength, measured as the force exerted by either all limbs or forelimbs of the mouse when gripping a pull bar assembly of a grip strength meter. WT and Gba mice did not display grip strength deficits, while SNCA tg mice developed age-related declines in grip strength. When Gba and SNCA mutations co-occur, as in Gba-SNCA mice, the grip strength deficits were exacerbated (Fig. 1B, Supp. Fig. 1B, Supp. Fig. 2F–G).

Healthy mice, when picked up by the tail and lowered towards a surface, extend their limbs reflexively in anticipation of contact. Mice with neurological conditions involving the motor cortex and spinal cord display hind limb clasping instead of extension. We tested the performance of mice for 30 seconds on this maneuver and quantitated the percentage of total time spent clasping (Fig. 1C). WT and SNCA tg mice did not show hind limb clasping. Gba mice showed a slight trend towards increased time spent clasping (Fig. 1C, Supp. Fig. 2H), whereas Gba-SNCA double mutant mice showed a significant increase across all ages, indicating a synthetic motor phenotype (Fig. 1C, Supp. Fig. 2H).

Open field behavior assay was performed to evaluate overall locomotory behavior. Distance traveled exploring an open box (Fig. 1D) was comparable among all groups across the four ages, except for Gba-SNCA mice. At 9 months, a significant loss in exploratory/locomotory behavior was noted for Gba-SNCA mice, which further worsened at 12 months compared to other groups (Fig. 1D, Supp. Fig. 2I). All genotypes did not exhibit anxiety-like behavior, as evaluated by the time spent in the inner and outer circles of the open field at 12 months of age (Supp. Fig. 1C). Additionally, there was no significant difference across the mice strains for body weight (Supp. Fig. 1J). However, Gba-SNCA double mutants stopped gaining body weight after 6 months of age, unlike other mice strains (Supp. Fig. 2J). In summary, motor behavior assessments demonstrate that Gba mutants do not exhibit any appreciable motor deficits. Nonetheless, presence of the Gba mutation significantly exacerbates existing age-related motor deficits in SNCA tg mice.

Next, we evaluated the impact of Gba and SNCA on cognition by employing fear conditioning and novel object recognition (NOR) tests. To avoid confounds due to learning, we performed these tests on two separate sets of mice at 3 months and 12 months, prior to and after the onset of motor behavior deficits in SNCA tg mice (Fig. 1A–D). For fear conditioning experiments, we initially habituated mice to standard operant boxes, followed by exposure to a paired neutral stimulus (a tone) and an aversive stimulus (a mild electric shock) on the training day. Cognitively normal mice associate this pairing and exhibit a conditioned fear response i.e. freezing when exposed to the tone alone on the testing day (24 hours later). We counted the number of freeze episodes after start of the tone and observed a conditioned fear response in WT and SNCA tg mice at both 3 and 12 months of age (Fig. 1E). However, Gba and Gba-SNCA mice did not show a significant conditioned fear response on the testing day, especially at 12 months of age. While this is suggestive of a cognitive impairment, the results were confounded by the heightened freezing response shown by Gba and Gba-SNCA mice for aversive stimulus on the training day (Fig. 1E).

To substantiate and corroborate the cognitive findings from fear conditioning, we performed a NOR test. The NOR test is routinely used in rodents to assess recognition memory. Here, mice were presented with two similar objects during a familiarization session. After 18–20 hours, one of the two objects was replaced by a novel object. Mice, being exploratory animals, spend more time with the novel object when cognitively normal; the time spent exploring the novel object is the measure of NOR (Fig. 1F). Gba mice spent significantly less time with the novel object compared to WT at 3 months and maintained this behavioral phenotype at 12 months, suggesting an early cognitive impairment (Fig. 1E). As Gba mice do not have motor problems, these results reflect actual memory impairments. Interestingly, SNCA tg mice did not show deficits in the NOR test (Fig. 1F), whereas Gba-SNCA do. Significantly, the performance of Gba-SNCA and Gba were comparable in the NOR test (Fig. 1F). Thus, our cognitive behavior assays indicate that the Gba mutation by itself can lead to cognitive impairment and the poor performance of Gba-SNCA mice in cognitive tests is likely driven by the Gba mutation.

### Gba mutation exacerbates cortical α-synuclein pathology of SNCA tg mice:

**Figure 2:**
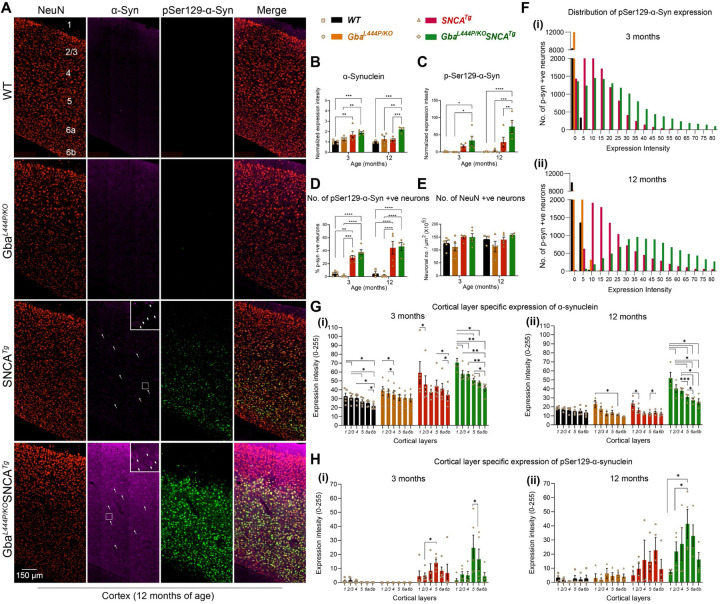
Gba mutation exacerbates α-synuclein pathology in the cortices of SNCA tg mice. **A.** Representative images of cortices of WT, Gba, SNCA tg, and Gba-SNCA mice (12 months) immunostained for NeuN (red), α-synuclein (magenta) and pSer129-α-syn (green). Note increased α-synuclein levels along with redistribution to neuronal soma (arrow) and pSer129-α-syn expression in the Gba-SNCA double mutant when compared to SNCA tg mice. **B.** Cortical α-synuclein expression at 3 and 12 months, normalized to respective WT average at each time point. **C.** Cortical pSer129-α-syn expression at 3 and 12 months, normalized to respective WT average at each time point. **D.** Percentage of cortical neurons positive for pSer129-α-syn at 3 and 12 months. **E.** NeuN positive cortical neuronal number at 3 and 12 months. **F.** Distribution of pSer129-α-syn expression intensity (Range of 0–255) in the cortical neurons at 3 months (i, WT: Mean = 0.35, 25% Percentile = 0.0024, 75% Percentile = 0.1252; Gba: Mean = 0.04, 25% Percentile = 0, 75% Percentile = 0.0058; SNCA tg: Mean = 13.05, 25% Percentile = 5.211, 75% Percentile = 18.534; Gba-SNCA: Mean = 23.781, 25% Percentile = 9.221, 75% Percentile = 33.972) and 12 months (ii), WT: Mean = 1.28, 25% Percentile = 0.439, 75% Percentile = 1.731; Gba: Mean = 1.482, 25% Percentile = 0, 75% Percentile = 2.124; SNCA tg: Mean = 26.282, 25% Percentile = 12.357, 75% Percentile = 32.834; Gba-SNCA: Mean = 53.741, 25% Percentile = 32.233, 75% Percentile = 66.691) of age. **G.** Cortical layer specific expression of α-synuclein at 3 (i) and 12 (ii) months of age. **H.** Cortical layer specific expression of pSer129 α-syn at 3 (i) and 12 (ii) months of age. n= 3–6, sex balanced. Data are presented as mean ± SEM. ns - not significant; *p < 0.05, **p < 0.01, ***p < 0.001, ****p < 0.0001. Scale = 150 µm

α-Synuclein aggregates, redistributes from presynaptic termini to the soma, and is phosphorylated at Ser129 in Lewy body pathology. To investigate whether Gba-mediated cognitive deficits and acceleration of motor deficits are associated with increased α-synuclein pathology, we performed immunohistochemistry on 3- and 12-month-old mice brains of the four genotypes, staining for α-synuclein, pSer129α-syn and the neuronal marker NeuN. We examined the expression levels and distribution of α-synuclein and pSer129α-syn, as measures of α-synuclein pathology. We found that Gba mice did not exhibit increased α-synuclein levels, redistribution or accumulation of pathological pSer129α-syn in the cortex both at 3 and 12 months (Fig. 2A–C). Similar observations were made in the CA1 hippocampus and by Western blotting of whole brain homogenates (Supp. Fig. 2B–I). These findings suggest that the Gba mutation alone is insufficient to cause widespread α-synuclein pathology in brain areas relevant for cognition, at these ages.

In Gba-SNCA mice, where GCase1 deficiency coexists with a pre-existing α-synuclein pathology, it results in significantly increased cortical α-synuclein and pSer129α-syn levels, especially at 12 months of age when compared to SNCA tg (Fig. 2A–C). We also noted increased redistribution of α-synuclein to the neuronal soma of Gba-SNCA mice as an independent measure of α-synuclein pathology (Fig. 2A, arrows, enlarged inserts). This exacerbation of α-synuclein pathology was confirmed by western blotting (Supp. Fig. 2G, I). Interestingly, in CA1 hippocampus, the expression of α-synuclein and pSer129α-synuclein, and the redistribution of α-synuclein to the neuronal soma in Gba-SNCA mice were comparable to SNCA tg mice (Supp. Fig. 2B–F). Thus, in contrast to the cortex, Gba mutation only nominally exacerbates pSer129α-syn pathology in synaptic layer of CA1 at 12 months (Supp. Fig. 2F). This might be in part due to high expression of the human SNCA tg in the hippocampus^27, 31^.

Next, we examined the intensity distribution of pSer129α-syn in the cortical neurons as well as cortical layer specific expression of α-synuclein and pSer129α-syn, without normalization to WT levels, at both 3 and 12 months of age (Fig. 2A, F–H). Gba mice did not show any pSer129α-syn pathology at both ages. Gba-SNCA mice had a higher intensity of pSer129α-syn pathology in cortical neurons at 3 months (median value of 20 vs 0 in WT and Gba, and 11 in SNCA tg mice). This was further increased at 12 months of age (Fig. 2F, i and ii) (median value of 47 vs 1 in WT, 0.2 in Gba, and 20 in SNCA tg mice). As the percentage of cortical neurons expressing pSer129α-syn in Gba-SNCA was comparable to SNCA tg mice (Fig. 2D), these data suggest that the neuronal burden of pSer129α-syn per cell in Gba-SNCA mice was greater. Importantly, though we observe severe α-synuclein pathology in SNCA tg and Gba-SNCA mice, we did not see cortical neuronal loss in any of the mice (Fig. 2A, E), indicating that the observed behavioral phenotypes are not due to gross neurodegeneration.

Analysis of layer-specific expression of α-synuclein revealed that cortical layer 1, which is heavily innervated by neurites and sparsely populated with neuronal soma, showed higher expression of α-synuclein compared to other layers, particularly layers 5 and 6, across all strains (Fig. 2G, i and ii, 3 and 12 months of age). Cortical layers 5 and 6a, which predominantly consist of excitatory neurons, showed higher pathological pSer129α-syn expression in SNCA tg and Gba-SNCA mice (Fig. 2H, i and ii, at 3 and 12 months of age). This increased expression of pathological α-synuclein in cortical layers 5 and 6a was more evident when pSer129α-syn expression was normalized to physiological α-synuclein (Supp. Fig. 2A, i and ii, 3 and 12 months of age). Together, these results suggest that the Gba mutation did not independently cause α-synuclein pathology at all ages but worsened pre-existing α-synuclein pathology in the cortex in SNCA tg mice, preferentially in layers 5 and 6a. When correlated with our behavior experiments (Fig. 1), these observations suggest that the cognitive deficits seen in Gba mutants emerge independently of pSer129α-syn pathology.

### Gba and SNCA driven cortical single nuclei gene expression changes:

To understand cellular diversity and transcriptional changes related to Gba and SNCA mutations and to obtain insights into the mechanisms for GBA-linked cognitive dysfunction, we performed single nucleus RNA sequencing (snRNA-seq) on cortical brain tissue from adult mice belonging to the four genotypes (n=14; 3–4 mice/genotype). We chose to perform this analysis on 12-month-old mice, as Gba-SNCA mice show enhanced behavioral deficits and α-synuclein pathology, while lacking gross neuronal loss or degeneration in the cortex, allowing us to investigate disease relevant mechanisms.

**Figure 3:**
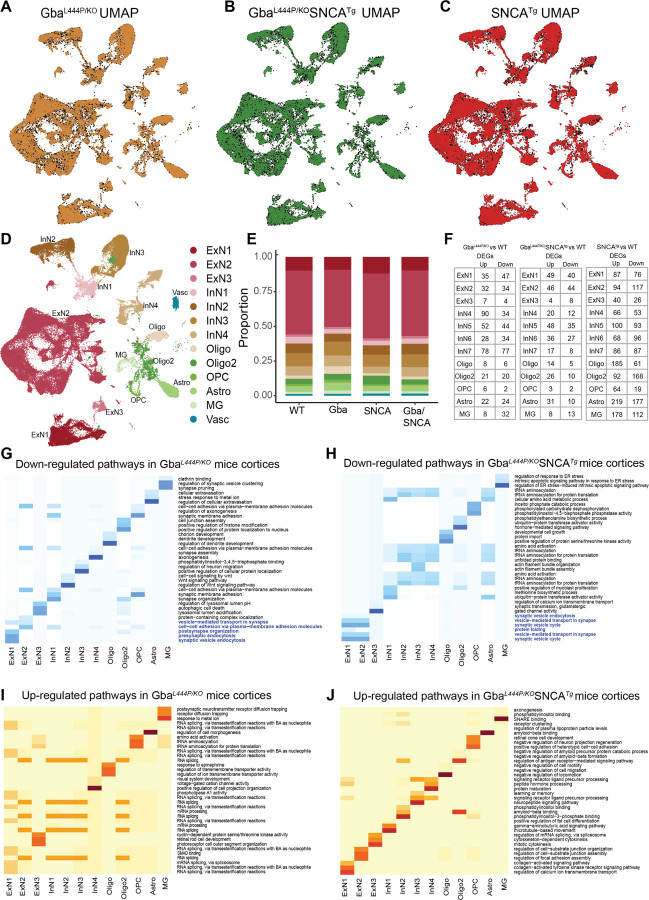
Cell type distribution, differential gene expression, and cellular pathway changes in cortex. Uniform Manifold Approximation and Projection (UMAP) dimension reduction for **A.** Gba (Amber), **B.** Gba-SNCA (Green) and **C**. SNCA tg (Red) overlayed over WT (Black) mouse cortical snRNAseq expression. **D.** UMAP showing clusters of cortical cell types identified by expression signatures. **E.** Proportions of the cell types in the cortices of wild type and transgenic mice. **F.** The number of differentially expressed genes (DEGs) per cell type in Gba, Gba-SNCA, and SNCA mutant mice after Bonferroni-correction for genome-wide comparisons and filtering out of genes with log2FC <I0.2I. **G-J.** Heatmap with the significantly down- and up-regulated gene ontology (GO) biological pathway alterations in 12 month old **(G, I)** Gba- and **(H, J)** Gba-SNCA mice, for each neuronal cluster type, revealed by unbiased analysis of enrichment of genome-wide corrected DEGs.

We dissected cortices and utilized our previous mouse brain nuclei isolation protocol^32^, followed by snRNA-seq on the 10X Chromium platform. After quality control, we recovered a total of 104,750 nuclei, including 31,906 nuclei from WT, 28,568 from Gba mutant, 26,579 from SNCA tg, and 17,697 from Gba-SNCA cortices. The mean reads per nuclei was 34,312 and the median number of identified genes per nuclei was 2410 in all samples. After cross-sample alignment, we performed clustering and recovered clusters that exhibited a spatial grouping in UMAP that were largely uncorrelated with individual samples or genotype (Fig. 3A–E, Supp. Fig. 3A, B).

The transcriptional signatures from the 104,750 nuclei, segregated well into 13 broad cortical cell type clusters (Fig. 3D, E). These cell clusters exhibited specific expression of established cell-type markers and grouped in a manner consistent with this expression (Supp. Fig. 3C). We identified all major cortical cell types, consisting of three types of excitatory neurons (ExN: ExN1, ExN2, ExN3), four types of inhibitory neurons (InN: InN1, InN2, InN3, InN4), two types of oligodendrocytes (Oligo and Oligo2), oligodendrocyte precursor cells (OPC), astrocytes (Astro), and microglia (MG) (Fig. 3D). Vascular endothelial cells (Vasc) were also identified but were not further studied. The characteristic marker gene expression for each cell cluster is shown in Supp. Fig. 3C–G. Expression in all ExNs is consistent with pyramidal neurons. In ExN1, differential expression (e.g., *Zfpm2*, *Dpp10*, *Frmpd4*, *Thsd7b*) was consistent with large layer 5 pyramidal neurons (e.g. Betz cells), while in the largest ExN subcluster, ExN2, (e.g., *Lingo2*, *Schip1*, *Cdh12*, *Unc5d*) differential expression was consistent with pyramidal neurons from several neocortical layers. Additionally, through characterization of expression of layer-specific marker genes (Supp. Fig. 3D–G), we identified that ExN1 largely consisted of layer 5 neurons, as shown by *Fezf2* expression (Fig. 3D, Supp. Fig. 3F). This was less true for ExN3. ExN2 exhibit signatures of ExNs from several cortical layers, including layer 2/3 and 4/5. The InN subclusters collectively express several classical InN markers, such as *Vip*, *Sst*, *Erbb4*, while the subclusters InN1 and InN3 specifically contain layer 2/3 interneurons (Supp. Fig. 3C). Typical marker signatures used for Oligo and Oligo2 (*Mbp*, *Ptgds*, *Mal*), suggests that Oligo2 also contains minor neuronal populations in addition to oligodendrocytes (Supp. Fig. 3C). The OPC (*Vcan*, *Epn2*, *Tnr*), Astro (*Aqp4*, *Prex2*, *Luzp2*) and MG (*Cd74*, *C1qa*, *Csf1r*) markers are consistent with prior literature^32, 33^. The relative proportions of major cell types were roughly similar between the four genotypes (Fig. 3E).

Next, we examined the expression of endogenous Gba, mouse Snca in WT brains and the expression of human transgenic SNCA (hSNCA) using Thy-1 in SNCA tg brains, in these clusters (Supp. Fig. 3H–J). Snca is enriched in excitatory neuronal clusters consistent with published literature (Supp. Fig. 3H)^34, 35^. Interestingly, Snca shows enrichment in ExN1 corresponding to layer 5 of the cortex^35^. This pattern was also true for hSNCA^35–37^, although hSNCA is also expressed in glial cell types in SNCA tg mice (Supp. Fig. 3H, I). The high hSNCA expression in the predominantly layer 5-populated ExN1 cluster (Supp. Fig. 3H) is consistent with the layer 5 specific increases of α-synuclein pathology demonstrated by immunohistochemistry (Fig. 2H, Supp. Fig. 2A). In contrast, Gba is generally found at low levels in neurons, oligodendrocytes, astrocytes, and microglia^38–40^ (Supp. Fig. 3J, i and ii).

After correcting for genome-wide comparisons, we identified up- and down-regulated differentially expressed genes (DEGs) in all cell types in Gba, Gba-SNCA and SNCA tg mice cortices compared with WT. The three genotypes showed DEGs in all cell types, with SNCA tg possessing the greatest number (Fig. 3F, Supp. Table 1–3). All DEGs significant after Bonferroni-correction for multiple comparisons were used in subsequent pathway analyses to understand the impact of these transcriptional changes, which is discussed below.

### Mutant Gba drives transcriptional downregulation of synaptic pathways in neurons:

To gain insights into the cognitive deficits seen in Gba mice, we focused on DEGs in neuronal clusters, comparing Gba with WT (Supp. Table 1). Strikingly, Gba mutant mice showed a general downregulation of many genes functioning at the synapse (*Arc*, *Syp*, *Actb, Nrg1*, *Nlgn1*, *Nrg1*, *Grm7*, *Grip1*, *Ptprd*, *Nlgn1*, *Il1rapl2*, *Gabra1*, *Cntn5*, *Lingo2*, *Erbb4*, *Nptn*, *Lrrtm4*, *Actb*, *Cntnap2*, *Lrfn5*), suggestive of a synaptic dysregulation signature related to Gba. *Ahi1*, a gene important for cortical development and vesicle trafficking was upregulated in all neuronal classes in Gba mice.

To define the major pathways impacted in Gba cortices, we performed unbiased gene ontology (GO) enrichment analysis comparing Gba to WT (Fig. 3G, I). As shown by the heatmaps depicting the top biological pathway changes, we found a consistent decrease in synaptic pathways in cortical ExNs of Gba mice (Fig. 3G). The large ExN clusters, specifically in the layer 5 containing ExN1, and the more mixed ExN2, shared robust downregulation of genes involved in SVE, presynaptic endocytosis, and vesicle-mediated transport in synapse (driven by reduced expression of *Syp*, *Actg1*, *Actb*, *Nlgn1*, *Grm8*, *Nrg1*, *Arc*) in Gba mice (Fig. 3G, highlighted, Supp. Table 4). Additionally, in Gba mice, ExN1 and ExN2 showed downregulation of cellular pathways and genes involved in both pre- and postsynapse organization (*Ctnnd2*, *Hspa8*, *Actb*, *Arc*), and synaptic protein-containing complex localization (*Nlgn1*, *Nrg1*, *Nptn*, *Dlg2*, *Arc*) (Fig. 3G, highlighted, Supp. Table 4). In the smaller ExN3 cluster, DEGs were fewer and involved in lysosomal lumen acidification (driven by reduced *Atp6v0c* expression) (Supp. Table 4). In contrast, the significant upregulated pathways in ExN1 and ExN2 of Gba involve RNA splicing (*Son*, *Prpf4b*, *Ddx5*, *Snrnp70*, *Srrm2*) (Fig. 3I, Supp. Table 4). Because Gba mice do not exhibit neuronal loss in the cortex at this age (Fig. 2E), these changes indicate synaptic dysfunction in Gba mutant mice, prior to gross neurodegeneration.

Inhibitory neurons (InN1-4) in Gba mutant mice showed downregulation of multiple synapse-associated pathways, including genes involved in synapse organization, synapse membrane adhesion (*Ctnnd2, Magi2, Actb, Mdga2, Adgrl3, Lrfn5, Lrrc4c, Il1rapl1* in InN1), synapse assembly (*Cntn5, Gabrb2, Pclo, Ptprd, Grid2, Lingo2, Erbb4* in InN4), Wnt signaling (*Ctnnd2*, *Nlk*, *Prkn*, *Cthrc1*, *Atp6ap2, Atp6v0c* in InN2), and axonogenesis (Chl1, Kif5c, Cdh11, Unc5c, Nfib in InN4) (Fig. 3G, Supp. Table 4), indicating an effect on synapse organization in these neurons. The upregulated pathways in InN1-4, similar to ExNs, are related to RNA splicing (Fig. 3I, Supp. Table 4).

Next, we extended this analysis to Gba-SNCA mice, comparing DEGs in neuronal clusters in Gba-SNCA with WT (Supp. Table 2). Interestingly, and consistent with our finding of a Gba-driven synapse effect, ExN clusters in Gba-SNCA mice also show downregulation of synapse related genes *Actb*, *Actg1*, *Unc13a*, *Cacna1a*, *Calm1*, *Btbd9*, *Prkcg*, *Pacsin1*) (Supp. Table 4). GO enrichment analysis comparing Gba-SNCA to WT revealed the top downregulated pathways in cortical ExN1 and ExN2 were synaptic vesicle cycle, vesicle-mediated transport in synapse, and SVE (Fig. 3H, highlighted). Although the individual DEGs between Gba and Gba-SNCA are not identical, the synaptic pathways being impacted are highly similar, suggestive of a common synaptic dysregulation signature related to Gba (Compare Fig. 3G with 3H). In Gba-SNCA InNs we see distinct pathways such as ubiquitin-protein transferase activator activity in InN1 and tRNA aminoacylation in InN2-4 being down regulated (Fig. 3H). The upregulated pathways in Gba-SNCA in ExNs are related to focal adhesion assembly and in InNs are diverse and include amyloid binding (Fig. 3J).

**Figure 4:**
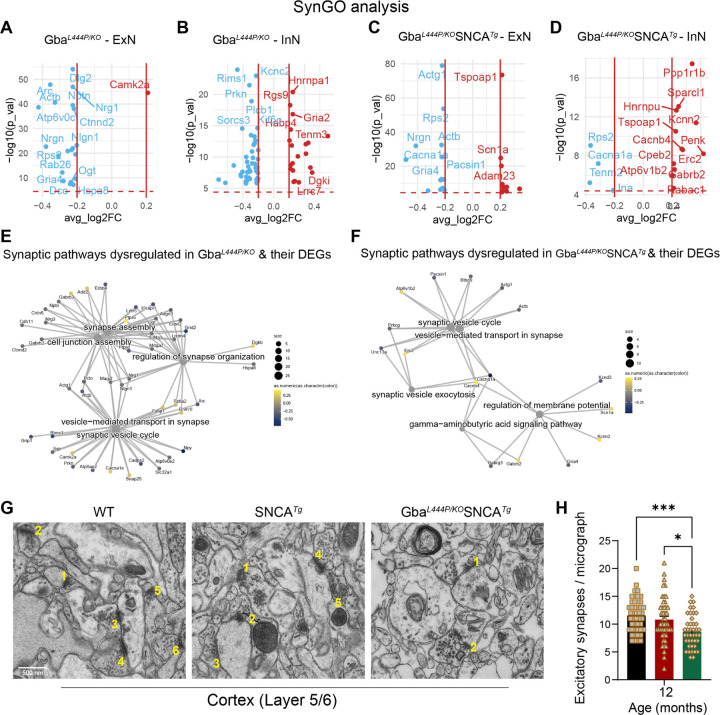
Analysis of synapse related gene expression in Gba and Gba-SNCA neurons shows similar deficits in synapse vesicle cycling. **A-D.** Analysis of significant DEGs that participate in synapse function, as annotated by SynGO, after Bonferroni-correction in excitatory (ExN1-3, **A, C**) and inhibitory (InN1-4, **B, D**) neurons. All genes with log2FC <0.2 were filtered out. **E, F.** Cnet plots of differentially expressed synapse associated genes as annotated by SynGO, in **(E)** Gba-, and **(F)** Gba-SNCA mice cortices, after Bonferroni-correction for multiple comparisons. **G.** Electron micrographs of cortical layer 5/6 excitatory synapses. Synaptic Vesicle denoted by arrow and clathrin coated vesicles by arrow heads. **H**. Quantitation of excitatory synapses in the cortical layer 5/6. Data are presented as mean ± SEM, Scale = 500 nm, *p<0.05, ***p<0.001., N=2 brains/genotype. 23–25 micrographs/genotype.

Next, we analyzed all neuronal DEGs through SynGO to define synaptic DEGs and get an accurate picture of the synaptic changes in Gba and Gba-SNCA mice. The SynGO analysis revealed more significant suppression of synaptic genes in ExN classes compared to InN classes in both genotypes (Fig. 4A–D). We visualized the significant differentially expressed synaptic genes in all neurons with Cnet plots, as annotated by SynGO, in Gba and Gba-SNCA mice cortices. These plots revealed enrichment of converging and predominantly down-regulated synaptic genes and pathways in both Gba and Gba-SNCA mouse cortices (Fig. 4E, F), consistent with the synapse assembly and synaptic vesicle cycle alterations demonstrated by GO analyses (Compare Fig. 3G, H, with Fig. 4E, F).

To evaluate if the downregulation of synapse organization pathways leads to a decrease in excitatory synapse number, we performed electron microscopy on 12-month old cortex samples. Electron microscopy was chosen as it allows for accurate quantification of synapse numbers while avoiding problems due to individual synaptic protein marker variability across genotypes. As seen in Fig. 4G and H, number of excitatory synapses in deep layers of cortex is indeed reduced in Gba-SNCA mice compared to SNCA and WT mice.

**Figure 5:**
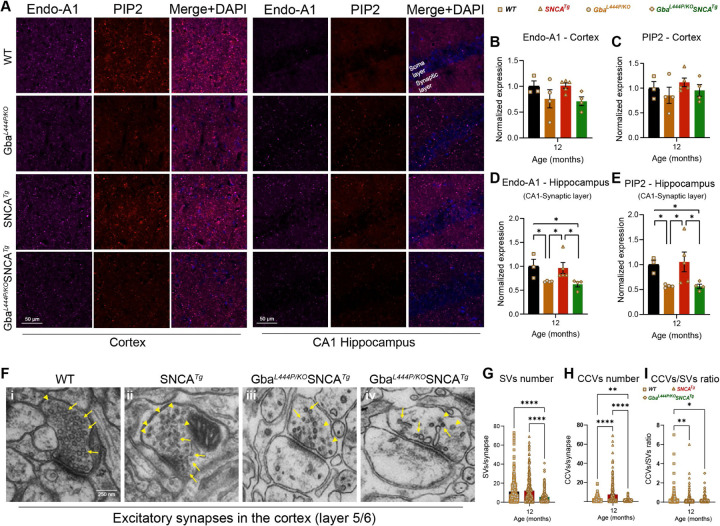
Decreased expression of SVE markers and loss of SVs in Gba mutants. **A**. Representative images showing cortical and CA1 hippocampal expression of endophilin-A1 (Endo-A1) and phosphatidylinositol 4,5-bisphosphate (PIP2), two markers of synaptic vesicle endocytosis, in WT, Gba, SNCA tg, and Gba-SNCA mice at 12 months of age. **B.** Cortical Endo-A1 expression at 12 months, normalized to WT average. **C.** Cortical PIP2 expression at 12 months, normalized to WT average. **D.** Endo-A1 expression in the CA1 Hippocampal synaptic layer, normalized to WT average. **E.** PIP2 expression in the CA1 hippocampal synaptic layer, normalized to WT average. Data are presented as mean ± SEM. Scale = 50 µm. * p<0.05. n=4–5 brains/genotype. **F.** Electron micrographs of excitatory synapses in cortical layer 5/6 showing SVs (arrows) and clathrin-coated vesicles (CCVs) in WT (**i**), SNCA tg (**ii**), and Gba-SNCA (**iii** and **iv**) mice. Note SVs with variable shapes and sizes in Gba-SNCA synapse **(iv). G-I**. Quantitation of SVs (**G**), CCVs (**H**) and their ratios (**I**) in the excitatory synapses of the cortical layer 5/6. Data are presented as mean ± SEM, Scale = 250 nm, *p<0.05, **p<0.01, ****p<0.0001, N=2 brains/genotype. 23–25 micrographs, 150–300 synapses, per genotype.

To validate SVE changes at the protein level, we immunostained WT, Gba, Gba-SNCA, and SNCA tg mice in cortex and hippocampus sections. We chose the SVE protein, Endophilin A1, as it is a risk allele for PD and the endocytic lipid PIP2. Conforming with the snRNA expression data, Endophilin A1 and PIP2, showed a decreased trend in the cortex (Fig. 5A, Cortex, B and C). Interestingly, in the synaptic layer in CA1 of the hippocampus where endophilin A1 and PIP2 are enriched, we noted significantly reduced Endophilin A1 and PIP2 expression in Gba and Gba-SNCA mice (Fig. 5A, CA1 hippocampus, D, E). Together, these observations corroborate our findings from snRNA-seq analysis of Gba-driven suppression of SVE genes and show these deficits are not limited to the cortex.

To determine whether these transcriptional and protein expression changes affect synaptic vesicle dynamics, we examined electron micrographs of excitatory synapses and quantified SVs and CCVs, which serve as proxies for SVE and SV recycling (Fig. 5F–I). In the Gba-SNCA mice, there was a marked reduction in both SVs and CCVs within the excitatory synapses of cortical layer 5/6, indicating a severe disruption of SVE and vesicle recycling (Fig. 5F–I). Interestingly, the SNCA tg mice displayed a significant increase in CCVs (Fig. 3F and H), consistent with findings in other α-synuclein models^41, 42^. Together, these findings reveal a distinct pattern of SVE disruption in Gba-SNCA mice, with reduced vesicle recycling efficiency, potentially leading to cognitive dysfunctions.

### ExN1 cluster contains vulnerable layer 5 cortical neurons:

As cortical layer 5 neurons exhibited the highest vulnerability in terms of α-synuclein pathology, (Fig. 2A, H, Supp. Fig. 2A), and transcriptional changes associated with SVE (Fig. 3G, H), we further investigated the ExN1 cluster which is enriched in these cells. ExN1 shows high expression of the hSNCA transgene as well as Gba (Supp. Fig. 3I, J). Upon subclustering, ExN1 was divided into six subclusters (Supp. Fig. 4A–D), all of which contained cells expressing the layer 5 marker *Fezf2* (Supp. Fig. 4D, E). One subcluster, ExN1.1, was characterized by high expression of Arc (Supp. Fig. 4D), which is downregulated in Gba mutant neurons (Supp. Fig. 4F). Our analysis confirmed greatest downregulation of synapse-associated genes in the major ExN1.1 subcluster in both Gba and Gba-SNCA mice (Supp. Fig. 4D, F–I). In contrast, non-synaptic DEGs were evenly up- and downregulated (Supp. Fig. 4F, G). Notably, synaptic vesicle cycle pathways were consistently downregulated in the major ExN1 subclusters, largely driven by the same genes identified in our non-targeted analysis (Fig. 3G, H). Additionally, Rab26, a key regulator of SV endocytosis and autophagy^43^, was downregulated in both Gba genotypes (Supp. Fig. 4H, I). These findings suggest that layer 5 excitatory neurons are selectively vulnerable because of both high hSNCA and Gba expression and synaptic deficits driven by Gba mutations, particularly SVE deficits. These mechanisms appear to act synergistically to exacerbate α-synuclein pathology in Gba-SNCA mice.

### Modest glial transcriptional changes in Gba and Gba-SNCA cortex:

Compared to neuronal clusters, glial clusters in Gba and Gba-SNCA cortices exhibited fewer DEGs (Fig. 3F, Supp. Table 1–3). In Gba, MG showed altered gene expression patterns indicative of reduced synaptic remodeling. Notably, synapse pruning (*C1qc, C1qa, C1qb*) and regulation of synaptic vesicle clustering (*Picalm, Magi2, Bcl2l1*) pathways were downregulated (Fig. 3G, Table 4). The upregulated pathways in Gba mutant MG are postsynaptic neurotransmitter receptor diffusion trapping (*Gphn*) (Fig. 3I, Supp. Table 4). These changes reinforce synapse dysfunction as a central pathological mechanism in Gba mutant cortex. The down and upregulated pathways in astrocytes in Gba are related to cellular extravasation and morphology (Fig. 3I, Supp. Table 4). In Gba, there are fewer DEGs in OPCs and Oligodendrocytes (Fig. 3F); therefore, clear pathway differences are harder to discern (Fig. 3G–I).

In Gba-SNCA, MG exhibit decreased endoplasmic reticulum stress response (*Ptpn1*, *Serinc3*), while SNARE binding (*Cacna1a, Picalm*) and aspects of phosphatidylinositol binding (*Thy1, Picalm*) were upregulated (Fig. 3H, J, Supp. Table 4). However, Gba-SNCA Astro exhibit decreased tRNA aminocylation (*Dalrd3, Lars2*) and increased amyloid-beta binding (*Apoe, Cst3, Clu, Prnp, Itm2c*) (Fig. 3H, J). In Gba-SNCA OPCs, phosphatidylinositol binding was downregulated (*Inpp4b*). while Oligodendrocytes showed modest pathway changes (Fig. 3H, J). Overall, in Gba mice, all glial cell types are muted in their responses, while in Gba-SNCA mice there are hints of activation in astrocytes. To confirm our analysis, we immunostained for microglia with the marker Iba1, CD68 for activated microglia, and GFAP for astrocytes (Supp. Fig. 5). We did not observe any significant increase in Iba1 or CD68+ve microglial number, suggesting negligible microglial activation in Gba and Gba-SNCA cortex. We observed increased GFAP levels in Gba-SNCA mice compared to WT, though it did not reach significance (Supp. Fig. 5A–D). Together, these data suggest that glial responses are modest, consistent with the snRNAseq data.

### Cortical transcriptional changes indicate broad synapse dysregulation in SNCA cortex:

SNCA tg mice showed the greatest number of DEGs compared to WT relative to Gba and Gba-SNCA mice (Fig. 3F, Supp. Table 3). The neuronal clusters showed broad alterations of synapse related gene expression. Both up and down-regulated DEGs are involved in synapse assembly, regulation of synapse structure or activity, and postsynapse organization (*Arc*, *Picalm*, *Hspa8*, *Actb*, *Ube3a*, *Nrxn3*, *Ptpro*, Lrrc4c, *Nlgn1*, *Mdga2*, *Lrfn5*, *Ctnna2*, *Fgfr2*, *Il1rapl1*, *Ppfia2*, *Lrrtm4*, *Dock10*, *Fgf13*, *Cntnap2*, (Supp. Figs. 6A–B, Supp. Table 3). SynGO analysis of these DEGs revealed regulation of synapse structure and function as the main pathway impacted in SNCA tg mice (Supp. Fig. 6C–D). As excitatory synapse number was not changed significantly in cortical regions of SNCA tg mice (Fig. 4G, H) compared to WT, these transcriptional changes are likely to result in functional deficits. Consistent with the observed cortical α-synuclein pathology at this age (Fig. 2A, D), unfolded protein handling was upregulated, as were pathways involved in protein folding and refolding (*Dnaja2*, *Hspa8, Hspa4, Hsp90aa1*) specifically in ExN1 and ExN2 clusters (Supp. Fig. 6B, Supp. Table 3). Consistent with this, regulation of protein ubiquitination was also upregulated (*Prickle1, Ubb, Hsp90ab1, Adgrb1, Sirt7, Hspa5, Hsp90aa1, Gps2* upregulation, with concomitant *Ube3a* downregulation) in all ExNs (Supp. Fig. 6B, Supp. Table 3). In glial cells, we see a wide range of changes. OPCs and oligodendrocytes show changes related to oligodendrocyte differentiation. In MG, immune receptor binding was the most reduced pathway and cation channel activity was increased (Supp. Fig. 6A–B, Supp. Table 3). In Astros, cell junction assembly was decreased, and ion channel activity was increased (Supp. Fig. 6A–B). However, we did not observe significant microgliosis or astrogliosis, as evaluated by immunohistochemistry, in the cortex of SNCA tg mice at this age (Supp. Fig. 5). In spite, of the widespread SNCA transcriptional changes, in Gba-SNCA cortices, the signatures of Gba are clearly evident.

## Discussion:

Surveys of PD and DLB patients and their caregivers highlight that maintaining cognitive abilities is one of their major unmet needs^44^. The *GBA* gene is an ideal choice to investigate this non-motor symptom, because it is the most common risk gene for PD^8^ and *GBA* mutations are linked to cognitive deficits in both PD and DLB^8, 18^. Here, we present detailed age-dependent behavioral and pathological phenotyping of the Gba-SNCA line alongside WT, Gba mutant mice, and SNCA tg model of PD. We demonstrate that Gba-SNCA mice recapitulate both cognitive dysfunction and motor deficits seen in GBA-linked PD and DLB. Significantly, we carried out cortical snRNA-seq analysis of these four mice genotypes and have built one of the first comprehensive *Gba* transcriptomic data sets. We encourage the scientific research community to utilize this rich dataset as a resource for generating hypotheses about PD and DLB.

### Gba-SNCA as a mouse model of *GBA*-linked PD and DLB.

Cognitive dysfunction has been noted in several existing models of PD designed to study motor deficits, including models that focus on α-synuclein pathology^45–48^. These models typically involve the overexpression of hSNCA mutations or the use of pre-formed fibrils (PFFs) to induce α-synuclein aggregation and propagation^48^. Such models have been instrumental in elucidating the mechanisms of α-synuclein pathology and its impact on neurodegeneration and cognitive decline. However, they often do not fully replicate the complex genetic and pathological landscape observed in human PD and DLB, and importantly, the contribution of *GBA*. Additionally, most Gba models are hampered by early lethality due to loss of ceramide and barrier function of skin, precluding age-related studies^27, 49^. Here, we build on our previous analyses of long-lived Gba mutant mice and Gba-SNCA^27^ and show that Gba-SNCA mice are an excellent model of GBA-linked PD and DLB. Gba-SNCA mice offer significant advancements as they exhibit worsened motor deficits compared to SNCA tg in an age-dependent manner (Fig. 1A–D) as well as cognitive deficits (Fig. 1E–F), closely mirroring the human condition. This is further evidenced by the exacerbation of cortical α-synuclein pathology in Gba-SNCA mice (Fig. 2). Most notably, by comparing Gba, SNCA tg and Gba-SNCA mice, we were able to demonstrate that the Gba mutation alone can drive cognitive dysfunction (Fig. 1E–F), while SNCA tg mice only exhibited motor deficits. These data agree with another recent study using heterozygous L444P Gba mutant mice which also showed cognitive deficits at 3 months of age^50^. Notably, Gba-SNCA mice exhibited enhanced motor deficits but cognitive deficits were on par with Gba mice. Congruently, Gba and Gba-SNCA mice show similar synaptic pathway deficits in neuronal populations as assessed by snRNAseq. In sum, Gba-SNCA mice capture the complexities of *GBA*-linked PD and DLB and serve as a good mouse model for these synucleinopathies.

### GBA-linked cognitive dysfunction is independent of α-synuclein pathology.

Our most striking finding is the observation that cognitive dysfunction occurs independent of or precede α-synuclein pathology in Gba mutants (Fig. 2). pSer129α-syn is the gold standard to define Lewy bodies in both PD and DLB^51–57 56, 58, 59^. Yet, we did not detect any pSer129α-syn in the cortex or entire brain of Gba mutant mice up to 12 months of age. Additionally, in Gba mutant mice, we did not observe redistribution of α-synuclein to the soma, a posttranslational modification independent measure of pathology or changes by western blotting (Fig. 2A, Supp. Fig. 2)^60^. This was most evident in the hippocampus, where the synaptic and cell body layers are segregated (Supp. Fig. 2B–F). While α-synuclein pathology in cortical areas does lead to cognitive dysfunction in mice overexpressing mutant α-synuclein or those injected with PFFs^48^, our study specifically challenges the necessity of α-synuclein pathology in the development of cognitive deficits in *GBA* mutations. Support for this comes from children with neuropathic forms of Gaucher disease, from *GBA* deficiency and show cognitive deficits but do not develop neocortical α-synuclein pathology^61, 62^. Still, it would be interesting to see if these mice develop features of Parkinsonism and α-synuclein pathology if aged beyond 12 months. Our findings also raise the possibility that other α-synuclein post-translational modifications or pathological forms could be involved in the disease process, warranting further investigation.

In contrast to cognitive deficits, motor deficits are strongly related to α-synuclein pathology as increased α-synuclein pathology in Gba-SNCA is clearly associated with worse motor performance. Notably, we observed significant α-synuclein pathology in cortical layers 5, similar to that seen in other PD mice models and human PD and DLB patients^35, 63^. Our snRNA-seq data suggest that layer 5 ExNs are also vulnerable to Gba mediated synaptic dysfunction, which could contribute to an increased accumulation of α-synuclein pathology in cortical layer 5, and in turn, the severity of the motor deficits seen in Gba-SNCA mice. Thus, Gba-SNCA mice also highlight specific cortical neuronal vulnerabilities, allowing for further investigations into cortical mechanisms of PD and DLB.

### Synaptic vesicle endocytosis and organization deficits in Gba-linked cognitive dysfunction.

Our snRNAseq analysis showed clear evidence of synaptic changes rather than the anticipated lysosomal gene changes. In fact, the only salient lysosomal changes were downregulation of *Psap* (in both ExN1 and ExN3) and *Atp6v0c* seen in all neuronal classes in Gba mutant cortex. *Psap* mutations, also known as *PARK24*, cause atypical Gaucher’s disease. *Psap* encodes Saposin C, which is an activator of Gba encoded GCase1 enzymatic activity^64^. *Atp6v0c* encodes the lysosomal V-ATPase that functions in lysosomal lumen acidification. In contrast, many synaptic genes that function in synaptic vesicle cycle, SVE, synapse organization, synapse membrane adhesion, and synapse assembly were downregulated in neuronal clusters in the cortex of Gba mutant as well as in Gba-SNCA mice (Fig. 3), suggesting common synaptic dysfunction mechanisms linked to Gba. Because Gba mutant mice do not show any significant α-synuclein pathology in the cortex at this age, this implies that synaptic dysfunction directly contributes to the observed cognitive deficits, rather than a consequence of disease pathology or neurodegeneration. In support of this tenant, we indeed observe excitatory neuronal synapse loss in cortex of Gba-SNCA mice (Fig. 3E–F). In other dementias such as Alzheimer’s disease, synapse loss correlates tightly with cognitive decline^65^. Our findings suggest that this is also true for GBA-linked PD and DLB, in line with available clinical studies^66–68^.

SVE was the major pathway downregulated in neurons in both Gba mutant and Gba-SNCA mice. Three key genes driving this pathway are *Hspa8*, *Dlg2 and Arc*. *Hspa8* (encoding HSC70) contributes to synapse vesicle uncoating and functions with *Dnajc6/PARK19*, a familial PD gene^29, 69^. *Dlg2* is a risk gene for sporadic PD that participates in receptor clustering in synapses^70^. Arc is an activity regulated gene that regulates transcription of many synaptic and SVE genes^71^. Interestingly, both clinical and experimental data link SVE deficits to cognitive deficits. The levels of dynamin1, the endocytic GTPase, to Lewy Body Dementia^72^. Additionally, patients with mutations in *DNAJC6/PARK19* and *Synj1/PARK20* which encode two key SVE proteins--auxilin and synaptojanin1—have cognitive deficits. Endocytic mutant mice show deficits in NOR and fear conditioning^73, 74^, supporting the tenet that SVE deficits lead to cognitive dysfunction. We suggest that Arc could serve as an upstream regulator of the synaptic transcriptional changes seen in Gba and Gba-SNCA cortices. Another contributor to the Gba-driven alteration of synaptic vesicle cycling is plasma membrane lipid composition changes in Gba mice. Emerging evidence indicates that altered sphingolipid composition, such as the one caused by Gba mutations, may interfere with phosphoinositide biology at membranes^75, 76^. Future research needs to address this important topic of co-regulated lipids beyond glucosylceramide.

### Conclusion/Future Prospects/Limitations:

Our study highlights the critical role of synaptic dysfunction in GBA-linked cognitive decline, in both Gba and Gba-SNCA mouse models. The Gba-SNCA mouse model effectively replicates both behavioral and histopathological characteristics of GBA-linked PD and DLB, offering a valuable tool for future research. Our findings reveal that GBA-linked cognitive decline occurs independent of α-synuclein pathology, challenging traditional views and highlighting the need to investigate alternative mechanisms of disease pathogenesis. While transcriptional analysis supported by histology revealed significant disruptions in SVE and synapse assembly, particularly in excitatory neurons, the upstream regulators of these transcriptional changes remain to be identified. While glial cells exhibit a more modest transcriptional response compared to neurons at this age, their involvement in synaptic dysfunction underscores the multifaceted nature of disease pathology. These insights emphasize the need for further research to explore the functional impact of these changes and their contributions to cognitive dysfunction in PD and DLB. Our comprehensive transcriptomic dataset provides a rich resource for exploring these further and we encourage the scientific community to leverage this data to further our understanding of GBA-linked disease mechanisms.

## Supplementary Material

Supplement 1

Supplement 2

Supplement 3

Supplement 4

Supplement 5
