# Supplementary material for: Synaptic vesicle endocytosis deficits underlie GBA-linked cognitive dysfunction in Parkinson’s disease and Dementia with Lewy bodies
Source: bioRxiv. 2025 Jan 2:2024.10.23.619548. Originally published 2024 Oct 23. Preprint. [Version 2] doi: 10.1101/2024.10.23.619548 (PMC11527026; doi:10.1101/2024.10.23.619548)
Supplement: Supplement 1 [file media-1.pdf]

Supplemental information:

Synaptic vesicle endocytosis deficits underlie GBA-linked cognitive dysfunction in Parkinson's disease and Dementia with Lewy bodies

DJ Vidyadhara[$,1,2,3,4], David Bäckström[$,1,2,5], Risha Chakraborty[1,2], Jae-Min Park[1,2,6], Jiapeng Ruan[3], Pramod K. Mistry[7], Sreeganga. S. Chandra[1,2,8*]

[1]Departments of Neurology and [2]Neuroscience, Yale University, CT, USA,

[3]Discipline of Neuroscience and [4]Center for Neurodegenerative Disease and Therapeutics, Chicago Medical School, Rosalind Franklin University of Medicine and Science, North Chicago, IL, USA

[5]Department of Clinical Science, Neurosciences, Umeå University, Sweden

[6]Van Andel Institute, MI, USA

[7]Department of Internal Medicine, Yale University, CT, USA

[8]Program in Cellular Neuroscience, Neurodegeneration and Repair, Yale University, CT, USA

* Corresponding Author

*Contact Information: sreeganga.chandra@yale.edu; Ph: +1-203-785-6172

[$]Contributed equally

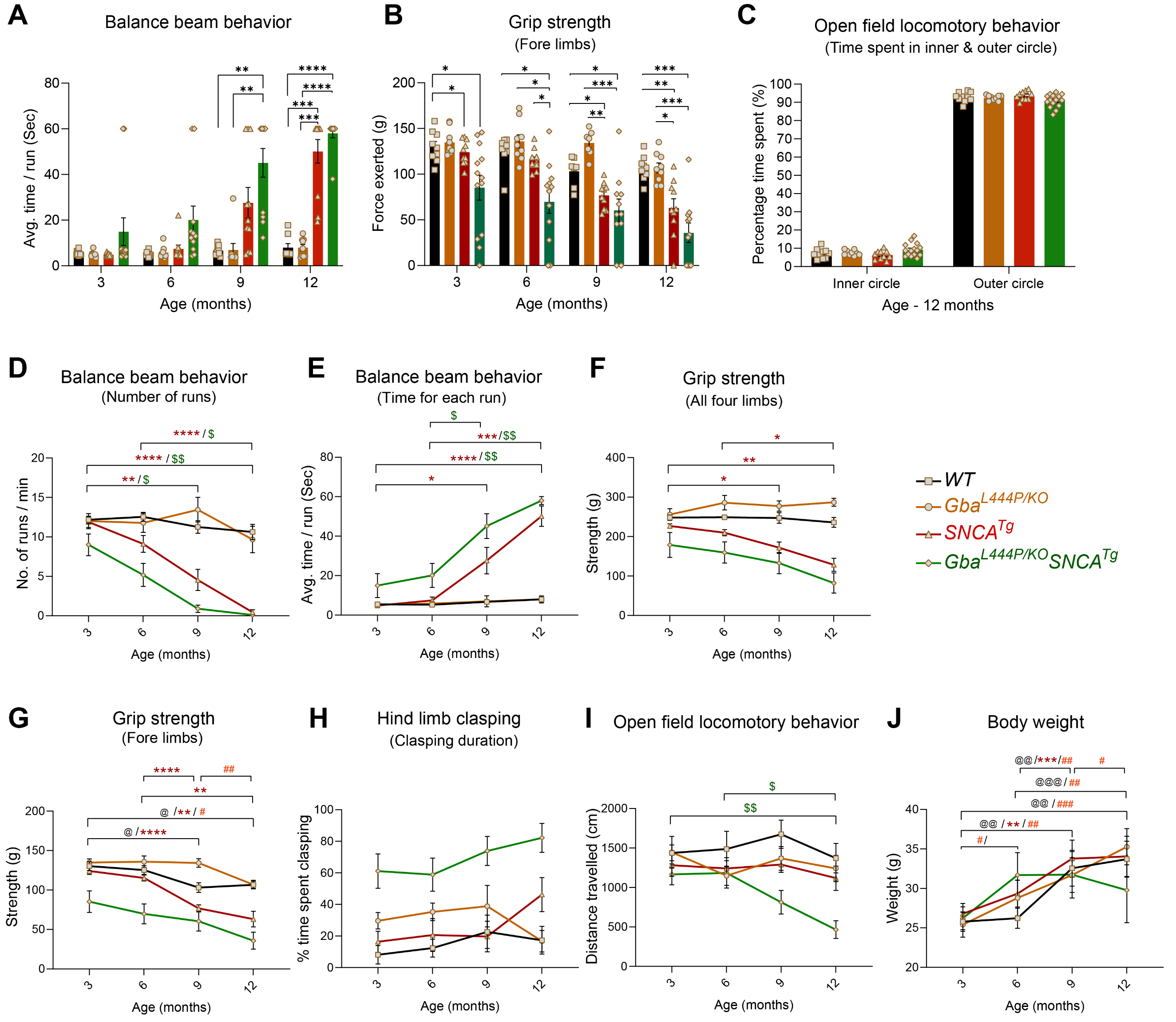

Supplementary Figure 1. Age-related progressive worsening of motor behavior deficits in SNCA tg and Gba-SNCA mice. Longitudinal motor behavior evaluations for WT, Gba, SNCA tg, and Gba-SNCA double mutant mice across 3, 6, 9 and 12 months of age. **A.** Balance beam behavior test showing time taken per each run. **B.** Grip strength of forelimbs. **C.** Time spent in inner and outer circle of an open field as a measure of anxiety-like behavior at 12 months of age. **D.** Balance beam behavior test showing age-related changes in number of runs per minute. **E**. Balance beam behavior test showing age-related changes in time taken per each run. **F**. Age related changes in grip strength (all four limbs). **G**. Age related changes in forelimb grip strength. **H**. Age related changes in hind limb clasping. **I**. Age related changes in open field behavior. **J**. Age related changes in body weight. n = 9-12 mice, sex-balanced cohorts. Data are presented as mean ± SEM. @ represent WT, # represent Gba mice, * represent SNCA tg mice, and $ represent Gba-SNCA double mutant mice. $^{@}p < 0.05$, $^{@@}p < 0.01$, $^{@@@}p < 0.001$, $^{@@@@}p < 0.0001$, $^{\#}p<0.05$, $^{\#\#}p < 0.01$, $^{\#\#\#}p < 0.001$, $^{\#\#\#\#}p < 0.0001$, $^{*}p < 0.05$, $^{**}p < 0.01$, $^{***}p < 0.001$, $^{****}p < 0.0001$ and, $^{\$}p < 0.05$, $^{\$\$}p < 0.01$.

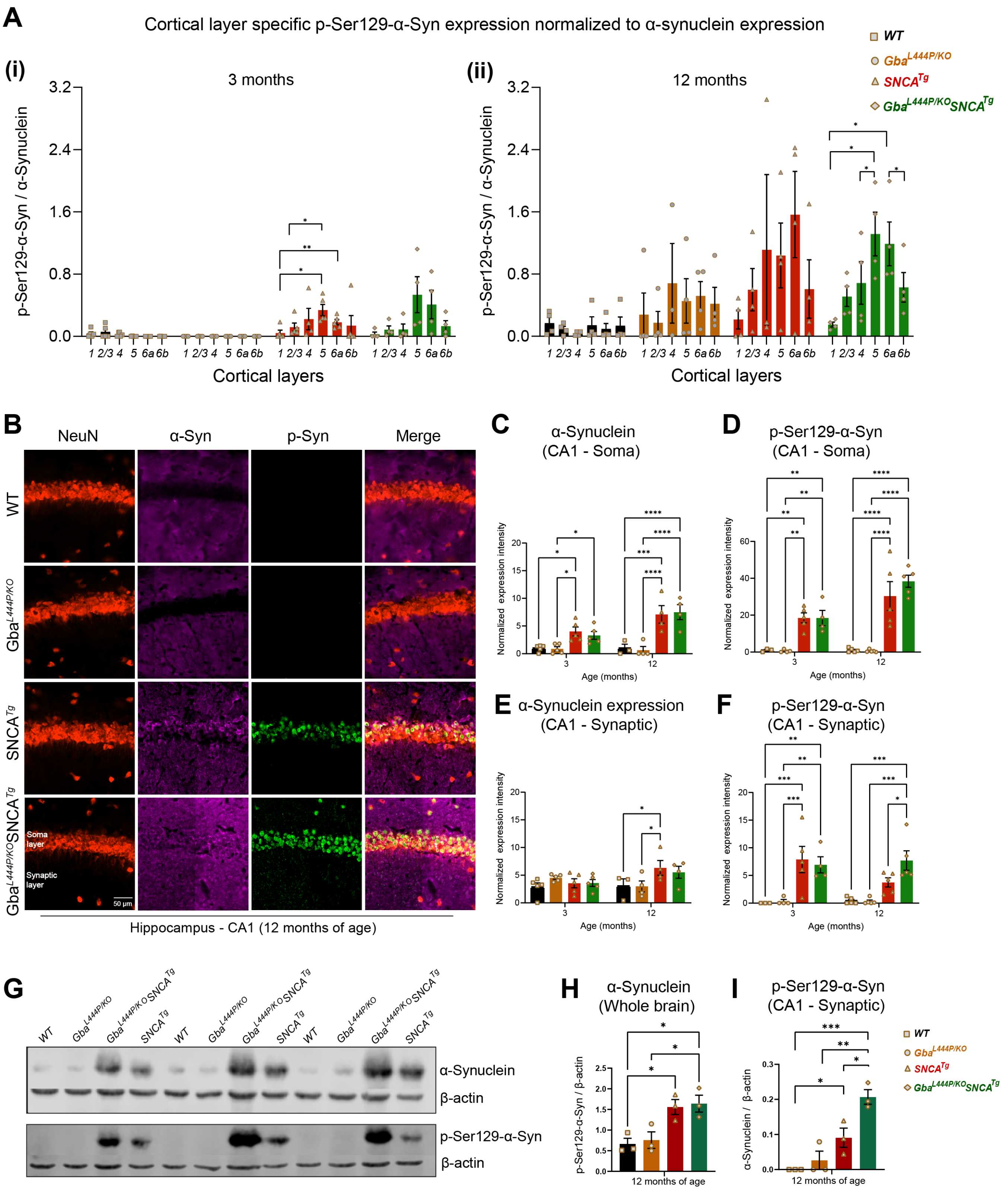

Supplementary Figure 2. α-Synuclein and pSer129-α-syn expression in cortex, hippocampus, and whole brain. A. Cortical layer specific pSer129-α-syn expression normalized to α-synuclein expression at the respective ages of (i) 3 and (ii) 12 months. **B**. Representative images showing α-synuclein and pSer129-α-syn expression in the CA1 subregion of the hippocampus in 12 month old mice. Scale = 50 µm. **C.** Quantification of α-synuclein expression in the soma layer of CA1 hippocampus. **D.** Quantification of pSer129-α-syn expression in the soma

layer of CA1 hippocampus. **E**. Quantification of α-synuclein expression in the synaptic layers of CA1 hippocampus. **F**. Quantification of pSer129-α-syn expression in the synaptic layers of CA1 hippocampus. **G.** Western blot showing α-synuclein and pSer129-α-syn expression in the whole-brain homogenates of WT, Gba mutant, SNCA tg, and Gba-SNCA tg mice at 12 months of age. **H.** Quantification of western blots for α-synuclein levels in whole brain. **I**. Quantifications of western blots for pSer129-α-syn levels in the whole brain. n = 3 -6 mice, equal number of males and females were used. Data are presented as mean ± SEM. $*p < 0.05$, $**p < 0.01$, $***p < 0.001$, and $****p < 0.0001$.

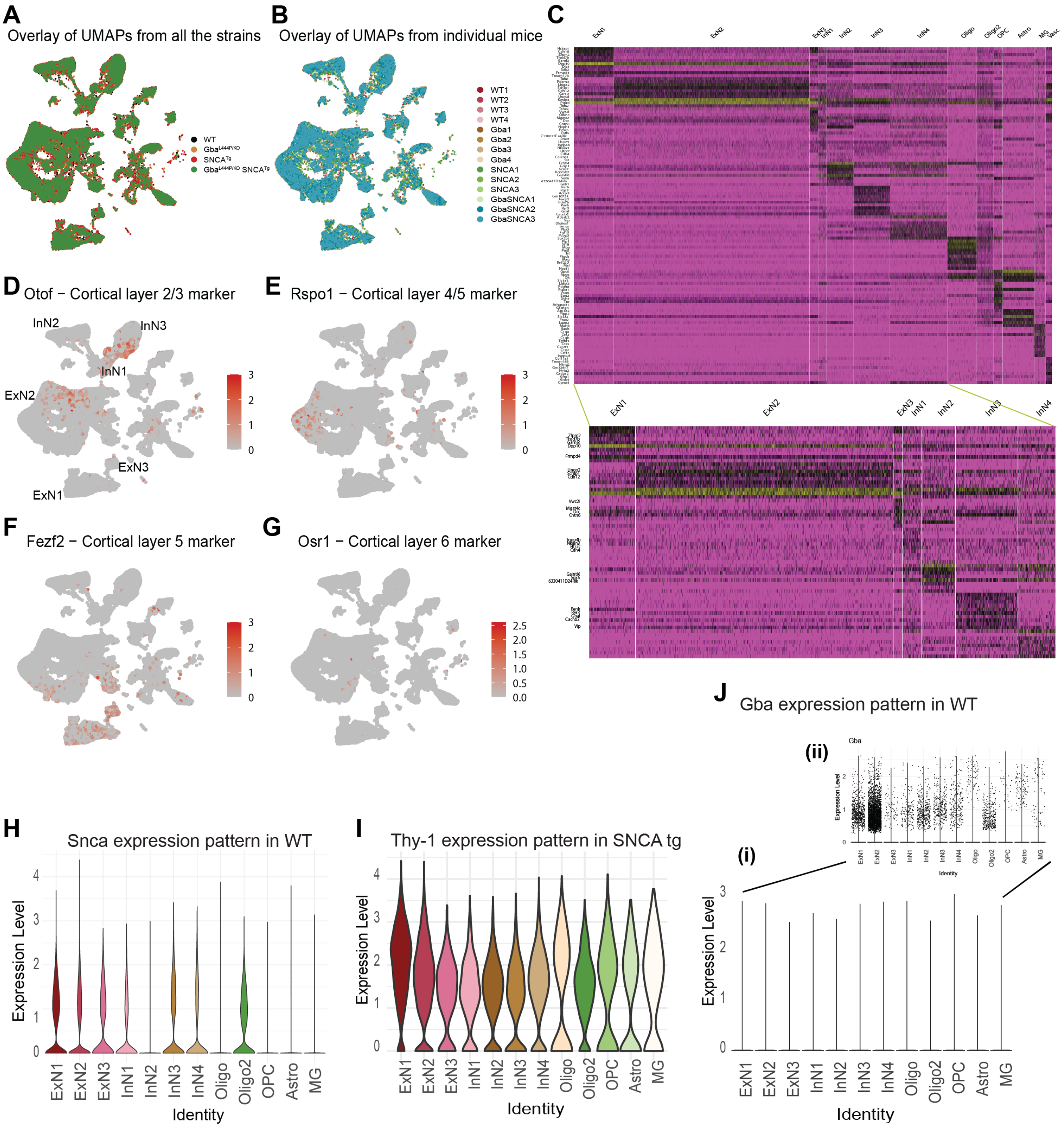

Supplementary Figure 3. UMAPs and expression of marker genes. A. Overlay of UMAPs for WT, Gba, SNCA tg, and Gba-SNCA showing lack of large-scale differences due to genotype. **B.** UMAPs for individual WT, Gba, SNCA tg, Gba-SNCA cortical samples. **C.** Cell type markers for the major clusters. **.** Cortical layer specific markers overlayed over UMAPs. Note, that Fezf2, the marker for Layer 5 neurons, labels ExN1 neuronal subcluster. **H.** Violin plots for expression of Snca in WT for denoted cell types. **I.** Violin plots for expression of Thy-1 in SNCA tg for denoted cell types. **J**. Violin (i) and dot (ii) plots for expression of Gba in WT for denoted cell types.

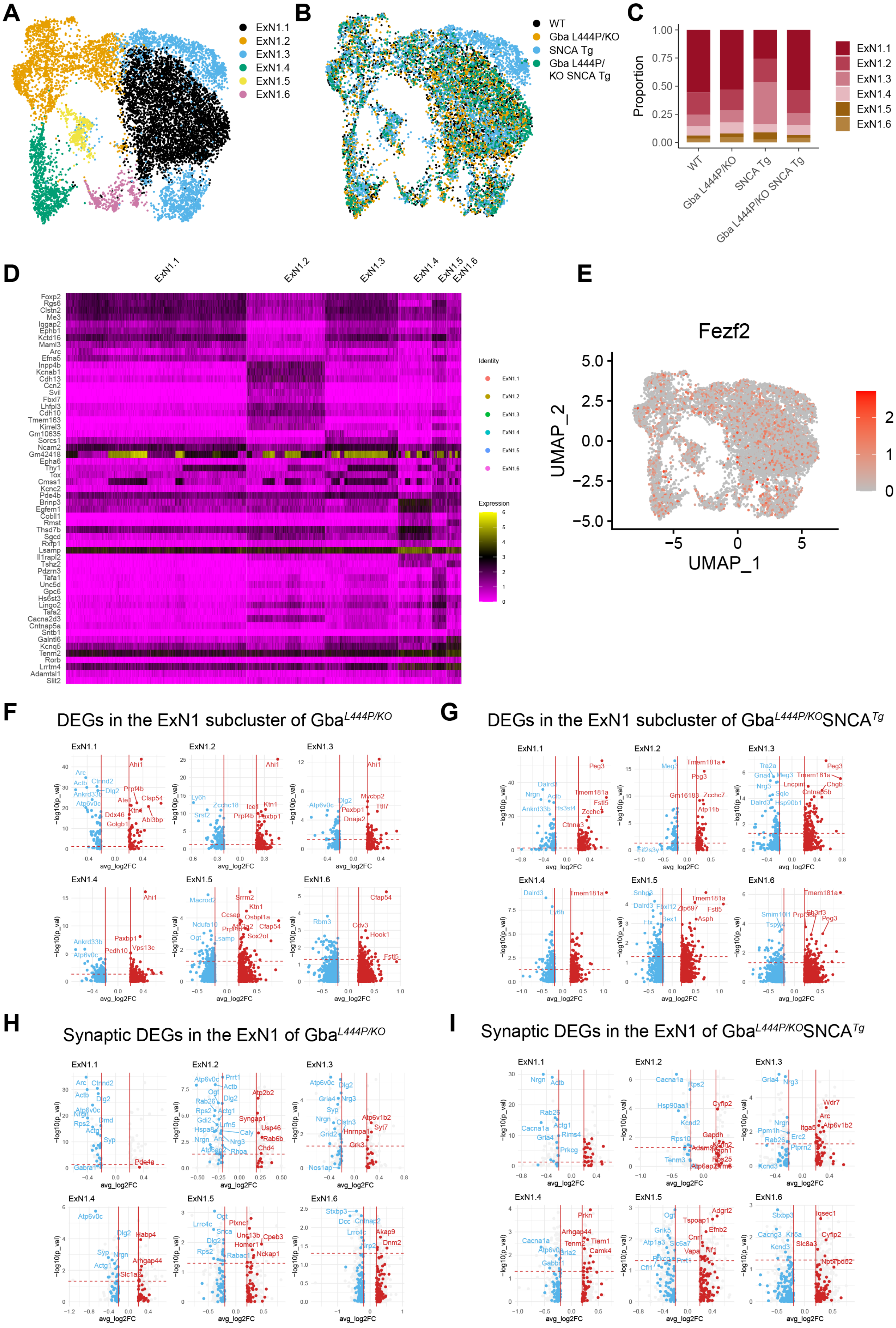
A
ExN1.1
ExN1.2
ExN1.3
ExN1.4
ExN1.5
ExN1.6
B
WT
Gba L444P/KO
SNCA Tg
Gba L444P/
KO SNCA Tg
C
Proportion
WT
Gba L444P/KO
SNCA Tg
Gba L444P/KO SNCA Tg
D
E
Fezf2
UMAP_1
UMAP_2
Identity
Expression
F
DEGs in the ExN1 subcluster of Gba^L444P/KO
G
DEGs in the ExN1 subcluster of Gba^L444P/KO SNCA^Tg
avg_log2FC
−log10(p_val)
H
Synaptic DEGs in the ExN1 of Gba^L444P/KO
I
Synaptic DEGs in the ExN1 of Gba^L444P/KO SNCA^Tg

Supplementary Figure 4: Cortical transcriptomic signatures of ExN1. A. UMAP showing the six subclusters found in ExN1. **B**. Overlay of the six subclusters of ExN1 in WT, Gba, SNCA tg, and Gba-SNCA samples. **C**. Proportion of cells in each ExN subcluster in the four genotypes. **D**, Heatmap showing characteristic cell type marker expression in the subclusters of ExN1. Note that high Arc expression is a characterizing marker trait of the largest subcluster ExN1.1. **E.** UMAP showing layer 5 marker Fezf2 expression pattern across ExN1 subclusters. **F.** DEGs found in the subclusters of ExN1 in Gba mice. **G.** DEGs found in the subclusters of ExN1 in Gba-SNCA mice. **H.** SynGO analysis showing synapse associated DEGs in ExN1 subclusters of Gba cortex. **I.** SynGO analysis showing synapse associated DEGs in ExN1 subclusters of Gba-SNCA cortex. Note that among all DEGs in ExN1 (**F** and **G**), DEGs are evenly distributed within up and down regulated DEGs, while the synapse associated DEGs (**H** and **I**) are selectively downregulated in all Gba as well as Gba-SNCA ExN1 subclusters. The downregulated DEGs involved in synapse vesicle cycle pathways were largely the same as shown in the main analyses, covering all cortical clusters in Fig. 4A-B and E-F, but in addition shows *Rab26* to be similarly downregulated in both genotypes (in ExN1.2 and ExN1.1, respectively).

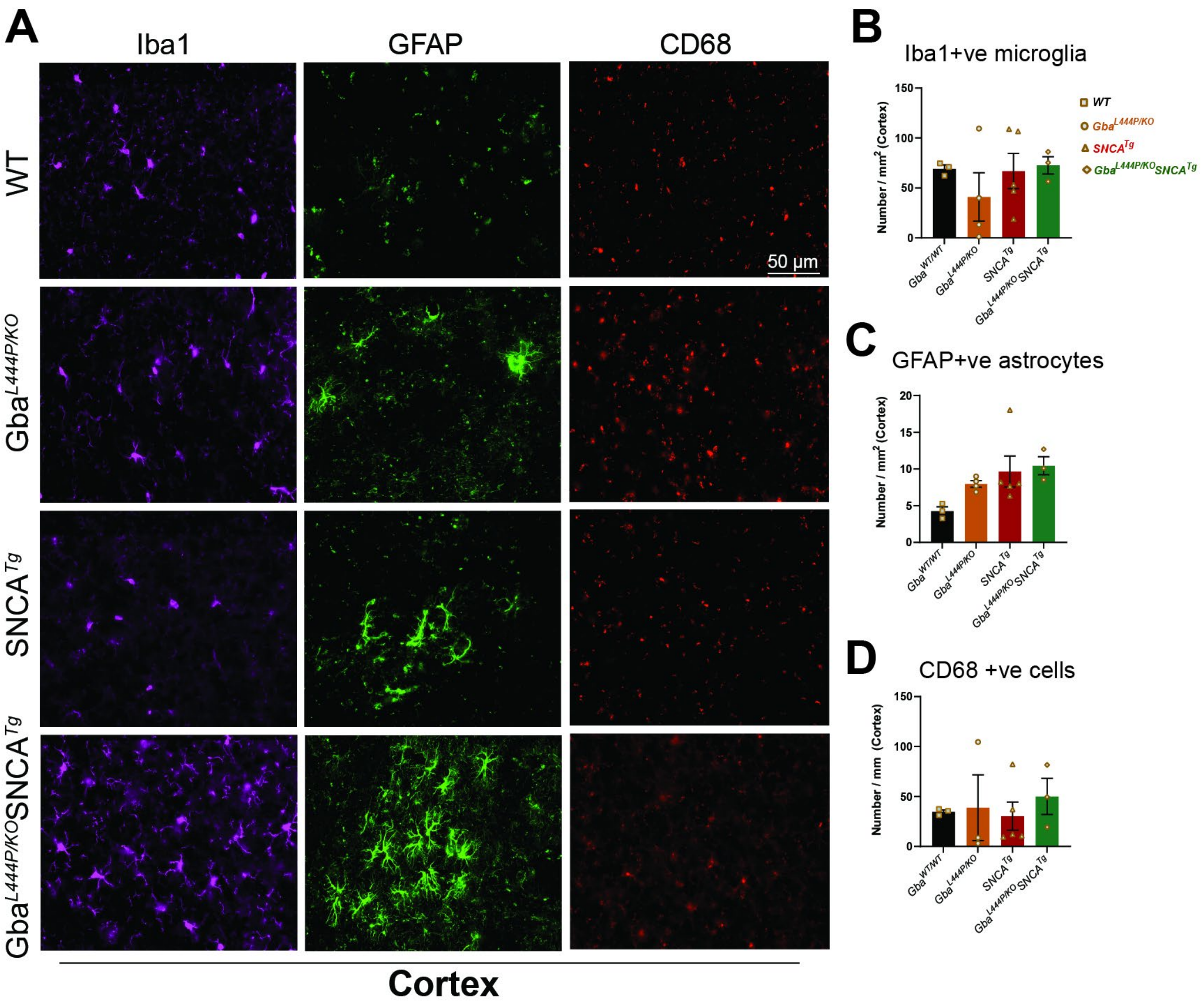

Supplementary Figure 5. Glial signatures in Gba and Gba-SNCA mice. A. Representative images showing cortical expression of Iba1, GFAP, and CD68 in WT, Gba, SNCA tg, and Gba-SNCA tg mice at 12 months of age. **B**. Number of Iba1 positive microglia in the cortex at 12 months. **C**. Number GFAP positive astrocytes in the cortex at 12 months. **D.** Number of CD68 (activated microglia marker) positive cells in the cortex at 12 months. Data are presented as mean ± SEM. Scale = 50 μm. * $p<0.05$, ** $p<0.01$. n=4-5 brains/genotype.

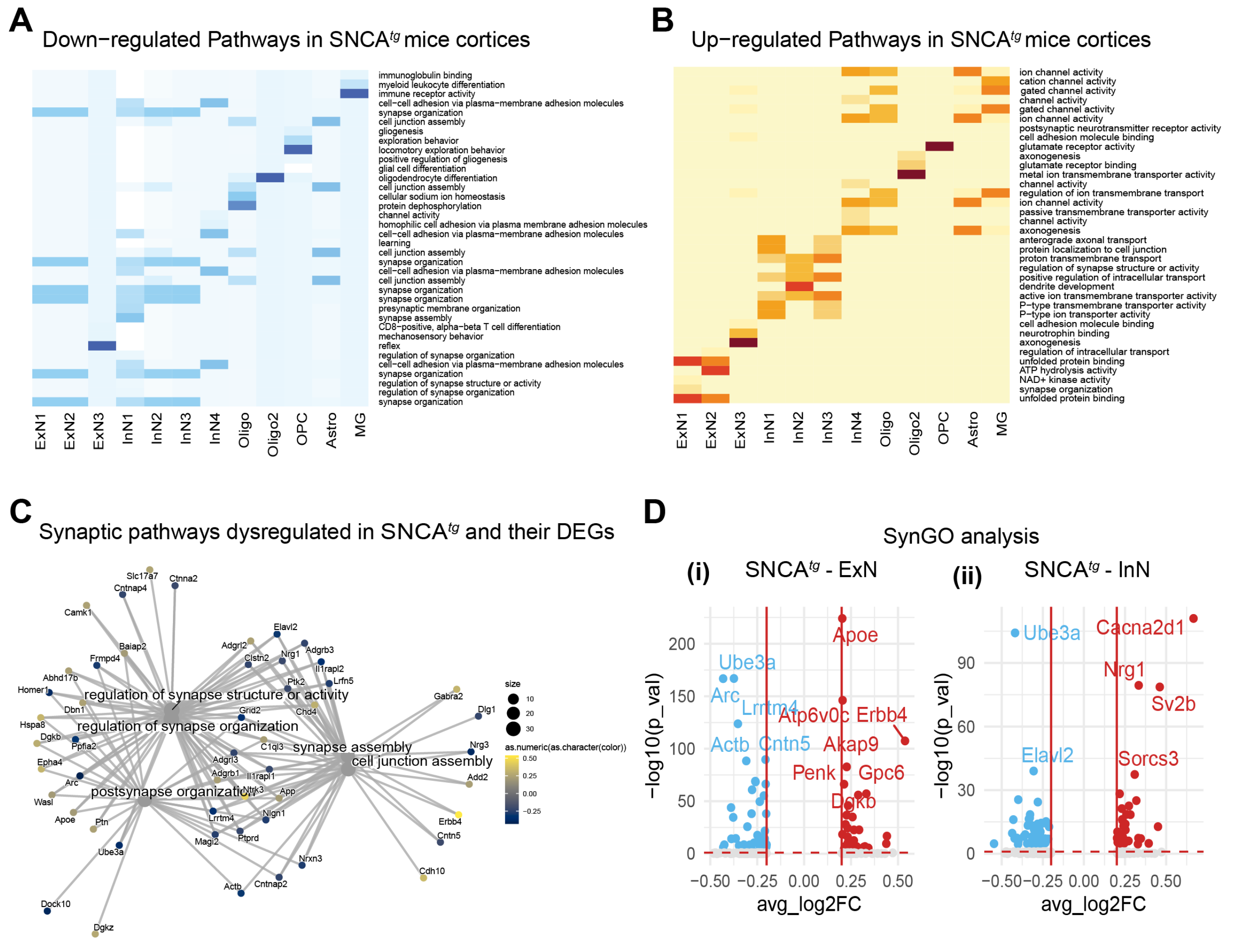

Supplementary Figure 6. Transcriptional signatures of SNCA tg cortex. **A-B**, Heatmap showing the most significant cortical gene ontology (GO) biological pathway alterations for each cell type, in 12-month old SNCA tg mice as revealed by unbiased analysis of enrichment of genome-wide corrected DEGs.. **C**. Cnet plot for synapse pathways with associated DEGs in SNCA tg mice **D**. SynGO analysis of DEGs in ExN and InNs of SNCA tg mice.
